# Cardiovascular and psychosocial risks among patients below age 50 with acute myocardial infarction

**DOI:** 10.1186/s12872-023-03134-w

**Published:** 2023-03-08

**Authors:** Åshild Faresjö, Jan-Erik Karlsson, Henrik Segerberg, Andrea Lebena, Tomas Faresjö

**Affiliations:** 1 Department of Health, Medicine and Care, General Practice, Linköping University, 581 83 Linköping, Sweden; 2 Division of Diagnostics and Specialist Medicine, Department of Health, Medicine and Care, Linköping University, 581 83 Linköping, Sweden

**Keywords:** Myocardial infarction, Cardiovascular risk factors, Psychosocial factors

## Abstract

**Background:**

Despite improvements in the treatment and prevention of cardiovascular disease since the 1960s, the incidence of cardiovascular diseases among young people has remained the same for many years. This study aimed to compare the clinical and psychosocial attributes of young persons affected by myocardial infarction under the age of 50 years compared to middle-aged myocardial infarction patients 51–65 years old.

**Methods:**

Data from patients with a documented STEMI or NSTEMI elevated acute myocardial infarction in the age groups up to 65 years, were collected from cardiology clinics at three hospitals in southeast Sweden. The Stressheart study comprised a total of 213 acute myocardial infarction patients, of which n = 33 (15.5%) were under 50 years of age and n = 180 (84.5%) were middle-aged, (51–65 years). These acute myocardial infarction patients filled in a questionnaire at discharge from the hospital and further information through documentation of data in their medical records.

**Results:**

Blood pressure was significantly higher in young compared to middle-aged patients. For diastolic blood pressure (*p* = 0.003), systolic blood pressure (*p* = 0.028), and mean arterial pressure (*p* = 0.005). Young AMI patients had a higher (*p* = 0.030) body mass index (BMI) than the middle-aged. Young AMI patients were reported to be more stressed (*p* = 0.042), had more frequently experienced a serious life event the previous year (*p* = 0.029), and felt less energetic (*p* = 0.044) than middle-aged AMI patients.

**Conclusions:**

This study revealed that persons under the age of 50 affected by acute myocardial infarction exhibit traditional cardiovascular risk factors like high blood pressure, and higher BMI, and were more exposed to some psychosocial risk factors. The risk profile of young persons under age 50 affected by AMI was in these respects more exaugurated than for middle-aged persons with AMI. This study underlines the importance of the early discovery of those at increased risk and encourages preventative actions to focus on both clinical and psychosocial risk factors.

## Introduction

Cardiovascular disease (CVD) has long been a steadily growing public health problem globally, with heart disease being the most common cause of death in the US in 2020 [1]. Death from CVD seemed to hit its peak in the US in the 1960s, and since there has been a decline in this number [1]. Furthermore, between 1990 and 2016 the disability-adjusted life years (DALY) adjusted by age decreased by 28.7%. Globally this indicates that the preventive work against CVD has been quite successful [2]. Although this has mainly been directed to the older population, there is still a lack of preventive measures for young myocardial infarction patients under the age of 55 [3].

The decline in mortality from CVD has largely been accomplished by a better understanding of the disease and its risk factors, and the development of more effective treatment and preventative actions. The approximately 47% decrease in mortality in the US between 1980 and 2000, was due to improved treatment and around 44% due to changes in risk factors [4]. In recent decades there has been an increased incidence of obesity and diabetes mellitus type 2 [4]. Recent research revealed that long-term stress with elevated cortisol concentrations before the onset of acute myocardial infarction (AMI) could be of importance, and the AMI could not fully be explained by only classical risk factors [5, 6].

Psychosocial and socioeconomic factors like socioeconomic status, occupation, education, and social support have not by themselves been shown to have a direct impact on mortality, but rather play a part in the context of developing depression and other risk factors. However, the feeling of low control has also by itself been associated with CVD [7–9].

Myocardial infarction before 46 years of age is quite rare and accounts for only approximately 10% of all male AMI cases, [10]. It is nonetheless a public health problem and because of the long remaining life expectancy of these patients, it should not be disregarded by preventative strategies. Despite this, a lack of awareness and a poorer understanding of risk factors has been suggested to be the reason for the standstill in incidence rates among young myocardial infarction (MI) patients [3]. The risk profile is yet to be fully understood, but according to previous studies, it mirrors that of middle-aged and older MI patients, with some exceptions. Classical risk factors seem to have an even greater impact on the risk of CVD for younger AMI patients [11]. This yields especially smoking, abnormal lipids, hypertension, and diabetes which have been shown to occur more frequently in young MI patients. Also they tend to be more exposed to some adverse psychosocial factors such as stress [12–16]. For younger persons in working life, anxiety disorders with symptoms like chest pain and breathlessness that resembles acute coronary syndrome could emerge [17]. Panic disorder has been suggested as a risk factor for cardiovascular disease and even as a trigger for acute coronary syndrome [18]. Myocardial infarction with non-obstructive coronary arteries (MINOCA) has been shown to occur more frequently in young MI patients as compared to older, with coronary spasm, structural dysfunction, and thrombotic disorders being some of the potential causes for MINOCA [19, 20].

Plaque rupture is the most common cause of MI in all ages [21]. An atherosclerotic plaque develops from a fatty streak, containing a deposition of low-dense lipoproteins (LDL)-particles and inflammatory cells, into an unstable plaque with an expanding necrotic core. When the plaque eventually ruptures it exposes the blood to the pro-coagulatory matrix, which causes the formation of a thrombus and might occlude the vessel [22]. However, the pathophysiological differences between younger and older MI patients are still largely unexplored.

The overall aim of this study was to explore the potential differences in cardiovascular risk profiles and psychosocial factors between younger and middle-aged patients affected by an acute myocardial infarction.

## Methodology

### Subjects

The study population was collected from three hospitals in southeast Sweden, one university hospital and two regional hospitals (but all three integrated into the university organization). Inclusion criteria were ST-segment elevation acute myocardial infarction (STEMI) or non-ST segment elevation acute myocardial infarction (NSTEMI), and an age of 65 years or younger. Exclusion criteria were not long enough hair (< 1 cm) on the vertex area, not speaking Swedish, or diagnosis of Addison’s disease or Cushing’s syndrome. The participants in the study were included when discharged from the hospital, in general, 2–3 days after the AMI. The participants were then asked to fill in a questionnaire and cut a piece of hair for cortisol measurement. After the patient´s written consent, some data from the medical records were also collected.

### Data collection

The questionnaire used was that of the STRESSHEART study which was composed of validated questions from the SCAPIS study [5, 23]. The participants answered questions about previous diseases and medication, as well as the heredity of MI and stroke. Perceived stress was measured on a Likert scale from 1 to 10, and participants got to answer questions about whether they had experienced a serious life event such as divorce, disease, or death in the family in the last year. There were also general questions about the patient’s psychosocial health, such as how they perceived their general health, and how often they felt calm, energetic, or sad. Some demographic and lifestyle factors were also collected, including smoking history, alcohol consumption, sleep habits, and physical exercise. Questions about socioeconomic like education, occupation, civil status, and ethnicity were also included.

From the medical records data was collected about the patient’s systolic and diastolic blood pressure, heart rate, weight, length, body mass index (BMI), and hip and waist circumference. The measurements SBP/DBP/ and heart rate were measured for all the AMI cases when discharged from the hospital clinic. A calculation of the mean arterial pressure (MAP) and the waist-hip ratio was also done. Hypertension was defined as previously diagnosed hypertension by a physician. Obesity was defined as a BMI ≥ 30 (kg/m^2^). The total number of classical risk factors was calculated for each patient; the risk factors were hypertension, obesity, hyperlipidemia, diabetes mellitus, current smoking, and heredity for MI or stroke. Heredity was defined as the incidence of AMI or stroke in first-degree relatives at any age. The inclusion day was used for the analysis of the seasonal onset of AMI. The cortisol concentration in hair was analyzed with an in-house radioimmune assay [24].

### Statistical analysis

**Fig. 1:**
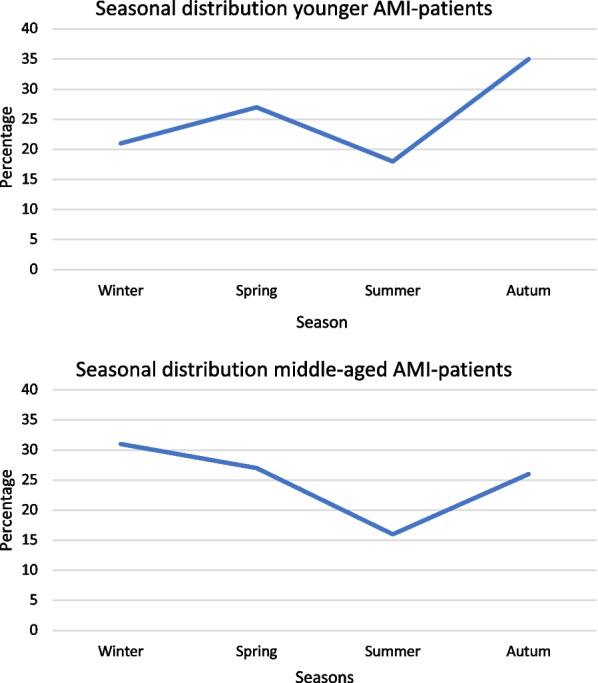
Comparisons of the onset in different seasons of myocardial infarction among AMI patients below age 50 (n = 33) and middle-aged (51–65 years) AMI patients (n = 180)

All analyses were performed using SPSS version 28. Descriptive statistics were presented using frequencies and proportions for categorical variables and means or medians for continuous variables. To compare frequencies for categorical variables chi-squared or Fisher’s exact test was used. To calculate the risk ratio, OR and 95% CI were applied. Cortisol concentration medians and IQR were compared using ANOVA. For some variables in Tables 2–4, there were some internal dropouts, but the calculation of the percentage was based on the actual number that answered the specific question. The population was analyzed based on their age at the onset of AMI. Young MI cases were defined as ≤ 50 years and middle age was defined as 51–65 years. Figure 1 was drafted using Excel. The significance level was set by a *p*-value < 0.05.

The study was approved by the Ethical Review Board at Linköping University (Dnr 2016-79-31, Dnr 2016-453-32, Dnr 2017-106-32). All participants in the study gave their written consent for participation.

## Results

A total of N = 213 AMI patients up to the age of 65, were enrolled in the study. Of these n = 33 was defined as younger (under the age of 50) and n = 180 as middle-aged (between 51 and 65 years) with a median age of 47 for the young group and 61 years for the middle-aged group. Females constitute 27.2% of the total AMI cases. Among the young AMI group, 30.3% were women, and 26.6% were females in the middle-aged group, see Table 1.

**Table 1:** The characteristics of the study population of younger AMI patients (below 50 years) and middle-aged AMI patients (51–65 years)

Variables	AMI patients below age 50		Middle-aged AMI patients 51–65 years	
	Female (n = 10)	Male (n = 23)	Female (n = 48)	Male (n = 132)
Age (median)	45.5	47	61	61
Waist circumference median (IQR)	96 (24)	101 (16)	93 (24)	102 (14)
Waist-hip ratio median (IQR)	0.89 (0.07)	1.01 (0.06)	0.91 (0.08)	1.01 (0.06)

Higher mean blood pressure could be seen in the young MI group when compared to the middle-aged, with systolic blood pressure 131 mmHg versus 124 mmHg; (*p* = 0.028), diastolic blood pressure 84 mmHg versus 78 mmHg; (*p* = 0.003) and mean arterial blood pressure 100 mmHg versus 93 mmHg; (*p* = 0.005). There was only a small difference seen in waist circumference and the waist-hip ratio between younger and older female MI cases, as shown in Table 1. Younger patients had a higher median BMI when compared to the middle-aged 29 versus 27; (*p* = 0.031). Obesity tends to be more common among the younger MI cases compared with older MI cases 39.4% versus 21.9%; (*p* = 0.055). The median cortisol concentration tends to be higher among the young AMI cases compared to older MI, however not significant (75.7 pg/mg vs. 70.2 pg/mg), see Table 2.

**Table 2:** Clinical measurements, and medical history for younger AMI patients below age 50 (n = 33) and middle-aged (51–65 years) AMI cases (n = 180)

Variables	AMI patients below age 50	Middle-aged AMI patients (51–65 years)	*p*-value
*Clinical values*			
Systolic blood pressure, mean (SD)	131 (19)	124 (17)	0.03
Diastolic blood pressure, mean (SD)	84 (12)	78 (11)	0.003
Mean arterial pressure, mean (SD)	100 (14)	93 (12)	0.005
Resting heart rate, mean (SD)	74 (12)	72 (12)	0.20
Length, mean (SD)	176 (10)	175 (9)	0.66
BMI, median (IQR)	29 (6)	27 (5)	0.03
Obesity, % (n)	39.4 (13)	21.9 (39)	0.05
Cortisol level, median (IQR)	75.7 (202.1)	70.2 (151.6)	0.7

Variables	AMI patients below age 50	Middle-aged AMI patients (51–65 years)	*p*-value
Medical cardiovascular History	% (n)	% (n)	
Earlier MI	18.8 (6)	27.5 (39)	0.27
Angina pectoris	6.3 (2)	11.9 (17)	0.36
Atrial fibrillation	0 (0)	2.1 (3)	0.40
Heart failure	0 (0)	3.5 (5)	0.27
Stroke	3.0 (1)	6.3 (9)	0.46
Hypertension	27.3 (9)	37.3 (53)	0.28
Hyperlipidemia	18.8 (6)	23.4 (34)	0.47
Percutaneous coronary intervention	9.4 (3)	18.9 (27)	0.17
Known hereditary MI	42.4 (14)	41.7 (75)	0.35
Known hereditary Stroke	24.2 (8)	35.1 (47)	0.24
Diabetes type 1	0 (0)	2.8 (4)	0.33
Diabetes type 2	15.2 (5)	12.0 (17)	0.62

There were not any significant differences in the patient’s medical history. However, heart failure, atrial fibrillation, and diabetes type 1 tended to be more common in the middle-aged group compared to the younger as well as tendencies of increased risk among the middle-aged cases concerning previous angina pectoris, stroke, and percutaneous coronary intervention, see Table 2. Cardiovascular medication in general like anticoagulants, anti-hypertensive, and anti-hyperlipidemia were more frequently reported among middle-aged MI patients, see Table 4.

For risk factors like smoking, no differences were seen, however, alcohol consumption was more frequent among the younger MI cases (35.3% vs. 20.9%). Physical activity was equal frequent between the two groups. Fewer sleep hours during the night as well as poor sleep quality were more common among the younger MI cases. A high level of perceived stress was also more pronounced among the younger MI group, see Table 3. Young patients were more likely to have experienced a serious life event in the last year (61.8% vs. 45.5%; *p* = 0.03). The young patients were also more prone to answer never or seldom when asked how often they felt energetic compared to the older MI cases (48.5% vs. 26.8%; *p* = 0.03). Low level of education was to some extent higher among the older as well as the number born outside Sweden. The estimated economic situation and occupation were quite equally distributed, except for the higher frequency of academics and chief positions among the middle-aged group, see Table 4.

**Table 3:** Comparisons of lifestyle risk factors between AMI patients below age 50 (n = 33) and middle-aged (51–65 years) AMI patients (n = 180)

Variables	AMI patientsbelow age 50		Middle-aged AMI patients (51–65 years)		*p*-value
	%	n	%	n	
Smoker yes	24.2	8	28.3	51	0.67
Drink alcohol					0.14
Rarely	39.4	13	42.4	75	
Sometimes	24.2	8	36.7	65	
Quite often	36.4	12	20.9	37	
Physical activity					0.96
Non/sporadic	57.6	19	60.8	107	
Regular	30.3	10	29.0	51	
Intensive regular	12.1	4	10.2	18	
Sleep habits					0.36
Poor/very poor	33.3	11	23.4	33	
Quite good	45.5	15	58.9	83	
Very good	21.2	7	17.7	25	
Sleep less than 7 h. night	54.5	18	38.3	54	0.08
Sleep more than 7 h. night	45.5	15	61.7	87	
Perceived everyday stress					0.22
Never	0.0	0	4.5	8	
Sometimes	60.6	20	68.5	122	
Always	39.4	13	27.0	48	
Perceived stress (median, IQR) rating (1–10) on a VAS scale	7 (3)		6 (5)		0.03

**Table 4:** Medications and psychosocial factors among AMI patients below age 50 (n = 33) and middle-aged (51–65 years) AMI patients (n = 180)

Variables	AMI patients below age 50		Middle-aged AMI patients (51–65 years)		*p*-value
	%	n	%	n	
Medications					
Antihypertensive	21.2	7	32.7	59	0.07
Anti-hyperlipidemia	9.1	3	18.3	33	0.13
Anticoagulants	0.0	0	11.1	20	0.03
Cardiovascular medications	3.0	1	18.3	33	0.02
Diabetes medications	12.1	4	11.7	21	0.70
Steroid based medications	15.2	5	15.6	28	0.79
Psychosocial factors					
Education					0.19
Low	12.1	4	25.7	46	
Medium	66.7	22	53.6	96	
High	21.2	7	20.7	37	
Occupations					0.88
Workers	54.5	18	53.5	92	
Civil servants	30.3	10	27.3	47	
Academic /boss	15.2	5	18.0	31	
Retired	0.0	0	1.2	2	
Ethnicity					0.05
Swedish	97.0	32	84.4	151	
Not Swedish	3.0	1	15.6	28	
Economy					0.90
Bad	18.2	6	18.5	33	
Good	81.8	27	81.5	145	
Civil status					0.50
Single	18.2	6	22.9	41	
Married/cohabited	81.2	27	77.1	138	
Encountered serious life events last year					0.03
Yes	60.6	20	41.5	73	
No	39.4	13	58.5	103	
How often have you felt calm?					0.78
Never/seldom	30.3	10	30.2	54	
Sometimes	36.4	12	29.6	53	
Most of the time	33.3	11	40.2	72	
How often have you felt energetic?					0.03
Never	48.5	16	26.8	38	
Sometimes	15.2	5	31.7	45	
Most of the time	36.4	12	41.5	59	
How often have you felt sad?					0.89
Never	66.7	22	65.5	93	
Sometimes	24.2	8	22.5	32	
Most of the time	9.1	3	12.0	17	

Some slight differences in the seasonal distribution on the onset of AMI could also be seen, both groups had a downward slope during the summertime. The younger had two peaks, one during spring and the second in autumn, and the middle-aged had their peak during the winter months, see Fig. 1.

## Discussion

### The main findings

The risks for cardiovascular disorders are generally increased with age. If younger people suffer a serious cardiac event there must likely be some explanations for their cardiac risk profiles. In this study, we found that younger person who suffers an acute myocardial infarction are more likely to have higher blood pressure, both systolic and diastolic, and they are also more likely to be obese than middle-aged MI patients. On the other hand, middle-aged were more likely to have heart failure and to use anticoagulants and cardiovascular medications. There were also some differences in psychosocial health, where the younger MI persons reported higher levels of stress, had more often experienced a serious life event last year, and reported themselves to be less energetic.

Younger MI persons having higher diastolic blood pressure are in accordance with previous findings in some other studies [13, 14]. According to a sub-study within the Framingham heart study, isolated diastolic hypertension is a better model for understanding cardiovascular risk than isolated systolic hypertension for young people under 50 years of age [13]. The present study found higher systolic hypertension, diastolic hypertension, and mean arterial pressure, for young MI patients. The higher mean hypertension and systolic hypertension in young patients might indicate that young MI patients are generally more burdened by high blood pressure than older MI patients, which may be because they are less medicated for hypertension [25].

The importance of obesity as a risk factor for cardiovascular diseases in a young population and how it differs from the general MI patient have been described earlier. Obesity has been shown to increase the risk of CVD in young populations [15]. In this study, BMI was higher in young MI patients compared to middle-aged MI patients, but no difference was evident in waist circumference or waist-hip ratio. This could indicate that BMI might be a better measurement for determining the cardiovascular risks in young people compared to middle-aged.

The risk of having previous cardiovascular diagnoses like angina pectoris, heart failure, percutan coronary intervention as well as a stroke, was two times higher among older MI patients as expected. No difference in the prevalence of diagnosed hypertension, hyperlipidemia, or diabetes mellitus was seen, but a slightly increased risk among the older group appeared. Most other studies defined heredity as the incidence of MI in a family member at an early age, below 55 years for male first-degree relatives or below 65 years for females [16, 25].

The classic type A behavior with high demands on themselves, impatient, and generally hostile and very competitive, tend to have a higher risk of coronary heart disease, which could not be explained by individual factors such as hypertension, diabetes, serum levels of cholesterol, or smoking [26, 27]. An increased cortisol concentration one month before MI, a measure of long-term stress, was seen among the younger study population, however not significant. The recovery prognosis after an acute myocardial infarction among younger patients has been shown to be related to financial barriers to health care [28]. Socioeconomic factors might play a role in functional recovery after myocardial infarction [29]). Chronic stress related to either job or marital strain was found to be associated with long-term adverse outcomes after acute myocardial infarction [30]. The presence of diabetes mellitus could increase the general risk of mortality after myocardial infarction, but young adults with diabetes mellitus experienced significant improvements [31].

Our findings also reveal that young MI patients are more likely to have experienced serious life events the year before the infarction and feel less energetic compared to middle-aged MI patients. There was no difference between age groups regarding experienced life events in previous years, indicating that a temporal aspect might be of importance. Since atherosclerotic lesions take many years to develop the idea that young MI patients have less coronary stenosis is not so farfetched, which has also been shown in previous studies [17, 18]. It can therefore be assumed that coronary occlusion is not the only cause of MI in these patients. This could indicate myocardial infarction with non-obstructive coronary arteries that there might be alternative causes for MI in young patients related to such as coronary spasms, structural dysfunction, and thrombotic disorders, but further studies are needed to understand the linkage to age [17, 18].

A seasonal difference in the onset of MI could also be seen in this study, where both groups had a decrease in the summer, while the older group had their peak during winter and the younger during autumn and even a small peak appeared during spring in this group. An earlier study of MI cases found a similar seasonal pattern, decreased occurrence from winter to autumn and from spring to summer. This was seen in both men and women, in different age groups, and in most geographic areas. In-hospital case fatality rates for AMI also followed a seasonal pattern, with a peak of 9% in winter [32]. This recent study almost followed the same pattern, but the younger ones had a more distinct peak in the autumn. Another recent study observed seasonal variation of incidence and in-hospital mortality and sex-specific differences regarding the seasonal variation of in-hospital mortality [33]. Seasonal variation in the onset of AMI could be affected by independent environmental and biological variables.

### Strengths and limitations

A major strength of this study is the number of variables included, which provides quite an extensive description of both the clinical and the psychosocial profile. The number of included AMI cases is however a limitation. The cross-sectional design of the study also limits the potential for truly understanding the association between cardiovascular risk factors and how these affect the risk for AMI in the younger. Many of the analyzed variables are based on self-estimations made by the participants, such as perceived stress, alcohol consumption, smoking habits, sleep, and exercise habits. All these retrospective questions based on individual perceptions could lead to some risks for recall bias. Especially questions about stress and self-estimated well-being can also have been affected by the myocardial infarction closely before their participation, mainly by making them remember the past as worse than it was, but this recall bias was minimized, since all participants filled in the questionnaire directly when they were discharged from the hospital, in general, only 2–3 days after the cardiac event.

## Conclusions

This study highlights the cardiovascular and psychosocial risk factors for young persons affected by myocardial infarction. Young MI patients are a group where little has improved over the years. The young patients protruded regarding higher blood pressure, higher BMI, increased self-reported stress, and even higher cortisol concentrations, they had more often experienced a serious life event and felt less energetic the year before the MI. These younger persons thereby follow a pattern where the traditional cardiovascular risk factors are evident before serious cardiac events. All these factors are preventable. This study underlines the importance of the early discovery of those at increased risk and encourages preventative actions to focus on both clinical and psychosocial risk factors.

## Data Availability

The dataset presented in this article is available upon reasonable request since it contains confidential information. Requests to access the dataset should be directed to the corresponding author (tomas.faresjo@liu.se).

## References

[1] Shields M, Haque A, de Ganzalez Barrington A (2022). Leading vauses of death in the US during the COID-19 pandemic, March 2020 to October 2021. JAMA Intern Med.

[2] Hay SI, Abajobir AA, Abate KH, Abbafati C, Abbas KM, Abd-Allah F (2017). Global, regional, and national disability-adjusted life-years (DALYs) for 333 diseases and injuries and healthy life expectancy (HALE) for 195 countries and territories, 1990–2016: a systematic analysis for the Global Burden of Disease Study 2016. Lancet.

[3] Gupta A, Wang Y, Spertus JA, Geda M, Lorenze N, Nkonde-Price C (2014). Trends in acute myocardial infarction in young patients and differences by sex and race, 2001 to 2010. J Am Coll Cardiol.

[4] Ford ES, Ajani UA, Croft JB, Critchley JA, Labarthe DR, Kottke TE (2007). Explaining the decrease in U.S. deaths from coronary disease 1980–2000. New Engl J Med.

[5] Faresjö T, Strömberg S, Jones M, Stomby A, Karlsson J-E, Östgren C-J (2020). Elevated levels of cortisol in hair precede acute myocardial infarction. Sci Rep.

[6] Stomby A, Strömberg S, Theodorsson E, Olsen Faresjö Å, Jones M, Faresjö T (2022). Standard modifiable cardiovascular risk factors mediate most of the association between elevated hair cortisol concentrations and coronary artery disease. Front Cardiovasc Med.

[7] Frasure-Smith N, Lespérance F, Gravel G, Masson A, Juneau M, Talajic M (2000). Social support, depression, and mortality during the first year after myocardial infarction. Circulation.

[8] Marmot MG, Bosma H, Hemingway H, Brunner E, Stansfeld S (1997). Contribution of job control and other risk factors to social variations in coronary heart disease incidence. Lancet.

[9] Mensah GA, Mokdad AH, Ford ES, Greenlund KJ, Croft JB (2005). State of disparities in cardiovascular health in the United States. Circulation.

[10] Doughty M, Mehta R, Bruckman D, Das S, Karavite D, Tsai T (2002). Acute myocardial infarction in the young-The University of Michigan experience. Am Heart J.

[11] Yang J, Biery DW, Singh A, Divakaran S, DeFilippis EM, Wu WY (2020). Risk factors and outcomes of very young adults who experience myocardial infarction: the partners YOUNG-MI registry. Am J Med.

[12] Yusuf S, Hawken S, Ounpuu S, Dans T, Avezum A, Lanas F (2004). Effect of potentially modifiable risk factors associated with myocardial infarction in 52 countries (the INTERHEART study): a case-control study. Lancet.

[13] Franklin SS, Larson MG, Khan SA, Wong ND, Leip EP, Kannel WB (2001). Does the relation of blood pressure to coronary heart disease risk change with aging?. Circulation.

[14] Taylor BC, Wilt TJ, Welch HG (2011). Impact of diastolic and systolic blood pressure on mortality: implications for the definition of "normal". J Gen Intern Med.

[15] Goliasch G, Oravec S, Blessberger H, Dostal E, Hoke M, Wojta J (2012). Relative importance of different lipid risk factors for the development of myocardial infarction at a very young age (</= 40 years of age). Eur J Clin Invest.

[16] Incalcaterra E, Caruso M, Lo Presti R, Caimi G (2013). Myocardial infarction in young adults: risk factors, clinical characteristics, and prognosis according to our experience. Clin Ter.

[17] Soares-Filho G, Arias-Carrión O, Santulli G, Silva A, Machado S, Valenca A, Nardi A (2014). Chest pain, panic disorder and coronary artery disease: a systematic review. CNS Neurol Disord Drug Targets.

[18] Soares-Filho GL, Machado S, Arias-Carrión O, Santulli G, Mesquita C, Cosci F (2014). Myocardial perfusion imaging study of CO(2)-induced panic attack. Am J Cardiol.

[19] Safdar B, Spatz ES, Dreyer RP, Beltrame JF, Lichtman JH, Spertus JA (2018). Presentation, clinical profile, and prognosis of young patients with myocardial infarction with nonobstructive coronary arteries (MINOCA): results from the VIRGO study. J Am Heart Assoc.

[20] Pasupathy S, Air T, Dreyer RP, Tavella R, Beltrame JF (2015). A systematic review of patients presenting with suspected myocardial infarction and nonobstructive coronary arteries. Circulation.

[21] Gulati R, Behfar A, Narula J, Kanwar A, Lerman A, Cooper L (2020). Acute myocardial infarction in young individuals. Mayo Clin Proc.

[22] Moore KJ, Tabas I (2011). Macrophages in the pathogenesis of atherosclerosis. Cell.

[23] Bergstrom G, Berglund G, Blomberg A, Brandberg J, Engstrom G, Engvall J (2015). The Swedish CArdioPulmonary bioimage study: objectives and design. J Intern Med.

[24] Mörelius E, Nelson N, Theodorsson E (2004). Salivary cortisol and administration of concentrated oral glucose in newborn infants: improved detection limit and smaller sample volumes without glucose interference. Scand J Clin Lab Invest.

[25] Oliveira A, Barros H, Azevedo A, Bastos J, Lopes C (2009). Impact of risk factors for non-fatal acute myocardial infarction. Eur J Epidemiol.

[26] Friedman M, Rosenman RH (1959). Association of specific overt behavior patterns with blood and cardiovascular findings; blood cholesterol level, blood clotting time, the incidence of arcus senilis, and clinical coronary artery disease. J Am Med Assoc.

[27] Rosenman RH, Brand RJ, Jenkins D, Friedman M, Straus R, Wurm M (1975). Coronary heart disease in Western Collaborative Group Study final follow-up experience of 8 1/2 years. JAMA.

[28] Beckman A, Bucholz E, Zhang W, Xu X, Rachel P, Dreyer R (2016). Sex differences in financial barriers and the relationship to recovery after acute myocardial infarction. J Am Heart Assoc.

[29] Alter D, Franklin B, Ko D, Austin P, Lee D, Oh P (2013). Socioeconomic status, functional recovery, and long-term mortality among patients surviving acute myocardial infarction. PLoS ONE.

[30] Arnold S, Smolderen K, Buchanan D, Li Y, Spertus J (2012). Perceived stress in myocardial infarction: long-term mortality and health status outcomes. J Am Coll Cardiol.

[31] Ding Q, Funk M, Spatz ES, Whittemore R, Lin H, Lipska KJ (2019). Association of diabetes mellitus with health status outcomes in young women and men after acute myocardial infarction: results from the VIRGO study. J Am Heart Assoc.

[32] Keller K, Hobohm L, Münzel T, Ostad MA (2019). Sex-specific differences regarding seasonal variations of incidence and mortality in patients with myocardial infarction in Germany. Int J Cardiol.

[33] Spencer FA, Goldberg RJ, Becker RC, Gore JM (1998). Seasonal distribution of acute myocardial infarction in the second National Registry of Myocardial Infarction. J Am Coll Cardiol.

